# Zwitterions fine-tune interactions in electrolyte solutions

**DOI:** 10.1073/pnas.2215585120

**Published:** 2023-02-14

**Authors:** James E. Hallett, Kieran J. Agg, Susan Perkin

**Affiliations:** ^a^Physical and Theoretical Chemistry Laboratory, Department of Chemistry, University of Oxford, Oxford OX1 3QZ, UK; ^b^Department of Chemistry, School of Chemistry, Food and Pharmacy, University of Reading, Reading RG6 6AD, UK

**Keywords:** liquids, electrolytes, electrostatics, confinement, forces

## Abstract

Cellular organisms regulate electrolyte composition in the cytosol to optimize intracellular molecular interactions at the same time as balancing external osmotic pressure. While osmotic pressure can be tuned using multiple ionic, zwitterionic, and nonionic solutes, interactions between proteins and other macromolecules are sensitive to the precise composition of the medium. Nonetheless, the roles of individual ions and nonionic solutes in mediating cellular interactions remain relatively unexplored, and standard buffer solutions used in laboratory studies often contain only a few simple salts. Here, we report on model experiments investigating the combined effect of ionic and zwitterionic solutes on interaction forces across electrolytes, revealing a clear role for zwitterions in modifying interactions compared to simple salt solutions. First, we find that zwitterions act to disrupt water layering at interfaces, leading to smoothed interaction potentials. Second, we find that zwitterions strengthen electrostatic repulsions by enhancing effective surface charge. Third, zwitterions enhance the effective dielectric permittivity of the solution, and this “dielectricizer” effect extends the range of electrostatic repulsions compared to solutions without zwitterion present. The latter two effects are likely important in stabilizing proteins and other macromolecules when external osmotic and mechanical pressure are very high and simple ionic solutes alone would lead to collapse.

## Significance

Electrostatic interactions underpin self-assembly, stability, and recognition between biological molecules. The natural environment mediating these interactions is complex, containing many ions, zwitterions, and polar components. Previously, it was assumed that the zwitterionic and polar osmolytes had the principal function of balancing external osmotic and mechanical pressure. By investigating their effect on the interaction between charged surfaces, we show that these osmolytes are far from being passive observers of electrostatic interactions: They play a significant role in defining the range and form of interaction potentials between charged particles.

The cellular interior is a dense milieu of diverse composition, with species ranging from small molecules to supramolecular structures. The organization, reactions, and interactions inside cells are controlled by mechanisms spanning wide time- and length-scales: Short-range solvophobicity drives self-assembly of amphiphilic lipids and proteins (1); liquid–liquid phase separation localizes biochemical reactions in membraneless compartments (2–4); and chemomechanical mechanisms drive ratcheting and locomotion (5). The exquisite balances involved in these processes arise from contributions to overall forces from electrostatic interactions, dispersion forces, steric hindrance (excluded volume), and specific interactions such as hydrogen bonds (6), all acting under nonequilibrium conditions due to chemical and thermal gradients. The identity and concentration of small molecules and ions of the surrounding medium are vital in this fine-tuning (7, 8). However, the diversity of this aqueous background—mirroring the chemical diversity in oceans and environments from which organisms evolved (9, 10)—presents a challenge for understanding the role of each component and synergies between them.

The ensemble of ions and molecules in the cellular space has a second, colligative, function: sustaining osmotic and mechanical pressure in balance with the external environment. Remarkably, cellular organisms thrive on the Earth in diverse environments with external salinity ranging from approximately 10^−3^ M (freshwater) to 5 M, e.g., the Dead Sea and the Great Salt Lake (11, 12) and external pressure ranging from approximately 0.08 MPa to 100 MPa, deep oceans (13). Any solute that binds or interacts favorably with water, and thus lowers the cellular water chemical potential, could be considered an osmolyte (14). Simplistically, osmolytes can be divided into ionic and nonionic osmolytes: Ionic osmolytes include small inorganic ions (Na^+^, K^+^, Mg^2+^, Ca^2+^, Cl^−^, HCO_2_^−^, SO_4_^2−^, PO_4_^2−^) as well as larger macromolecules with nonzero net charge; nonionic osmolytes include zwitterions and other molecules with zero net charge.

**Fig. 1.**
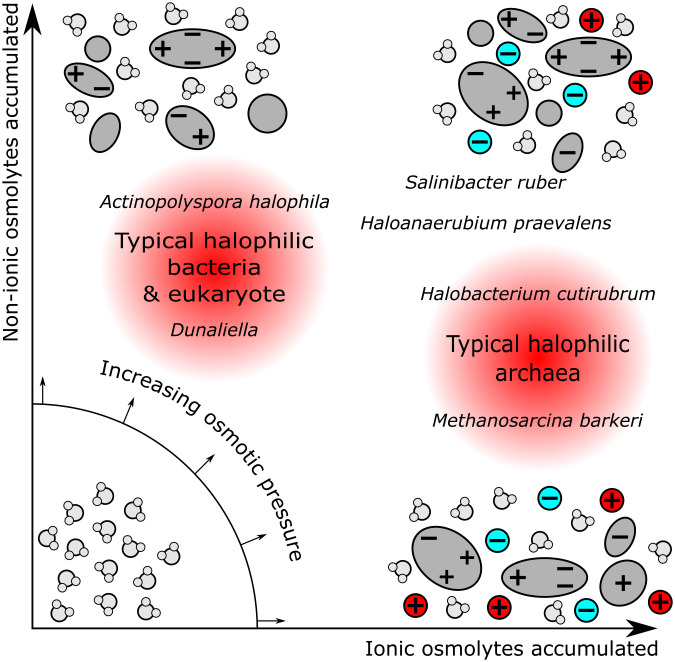
Schematic view of the osmoprotective strategies exhibited by halophilic organisms. Osmotic pressure can be adjusted by accumulation of ionic solutes (e.g., inorganic salts) and/or nonionic species (e.g., amino acids, and polyols). Examples are shown of classes and species which are known to adopt particular strategies. Halophilic archaea such as *Halobacterium cutirubrum* and *Methanosarcina barkeri* (15) tend to accumulate ionic solutes, although there are examples of bacteria which also adopt this method, such as the extremely halophilic bacteria *Salinibacter ruber* (16) and *Haloanaerobium praevalens* (17, 18), which exhibit intracellular salt concentrations similar to the growth medium. Moderately halophilic bacteria, e.g., *Actinopolyspora halophila* (19), fungi, and algae, e.g., *Dunaliella* (12, 20) instead accumulate nonionic osmolytes such as TMG and TMAO.

Various strategies have evolved to achieve the required osmotic balance, or more commonly an outward turgor pressure, through accumulation of ionic and nonionic osmolytes to maintain overall osmotic balance, as summarized in Fig. 1. Extremely halophilic organisms, particularly archaea (15), tend to accumulate high levels of simple ionic species including K^+^ and Cl^−^. In contrast, less extreme halophiles including various bacteria, fungi, and algae tend to accumulate small organic osmolytes such as the zwitterions trimethylglycine (TMG) or trimethylamine N-oxide (TMAO) (19, 21), polyols (in fungi and algae), and ectoine derivatives (in bacteria) (22). Furthermore, some zwitterionic solutes, in particular TMAO, provide resistance against hydrostatic pressure and are accumulated in organisms living in the deep oceans; these have also been termed “piezolytes.” The accumulation of ionic and of nonionic solutes, also called “salt-in” and “osmolyte-in” mechanisms, respectively, present different biochemical challenges. The former requires adaptation of the genome to maintain protein structures resilient to high ionic strength, frequently comprising an increase in the proportion of acidic amino acid residues expressed in the organism’s proteome in preference to basic residues (15). In contrast, the latter mechanism does not require such widespread genomic adaptations but can instead require a significant expenditure of energy in order to perform biosynthesis or selective transport from the environment of the desired compatible solutes. In higher organisms, more sophisticated mechanisms of osmoprotection and osmoregulation have developed, such as the presence of skin to sustain osmotic imbalance and the ability to vary urine concentration. Nonetheless, tissues exposed to locally high osmotic pressure, such as the renal medullary cells in the kidney, accumulate osmolytes to maintain function (23, 24).

The challenge of optimizing simultaneously for delicate interparticle forces and for osmotic pressure clearly demands a multicomponent osmolytic environment. However, the roles of individual osmolytic components and the synergies between them have been explored in relatively few studies. In particular, the assumption that nonionic osmolytes do not significantly modify interparticle forces has only recently begun to be interrogated (25–27). In the present work, we focus on a simple test case: We compare interaction forces measured across aqueous electrolyte solutions containing salt (KCl) and zwitterions (trimethylglycine, TMG) at varying concentrations. We report direct measurements of the interaction force between smooth negatively charged surfaces as a function of their separation distance across a range of electrolyte compositions, interrogating the effect of zwitterions on the overall interaction. Our measurements span a wide range of biologically relevant salt and zwitterion concentrations. We first study solutions containing only zwitterions, then only salt, to guide our interpretation of the effects resulting from their combination. We find that zwitterions have the effect of increasing the magnitude and range of repulsions between charged surfaces, implying a stabilizing effect on particles in solution. At sufficiently high zwitterion concentrations, distinct monolayers and multilayers of zwitterions build up at the surfaces. With salt also present, the water, ions, and zwitterion compete to determine the composition and structure of the near-surface region. Depending on their relative concentration, a range of distinct behaviors are possible; zwitterions tune the interactions relative to salt-only electrolytes.

## Experimental Summary.

**Fig. 2.**
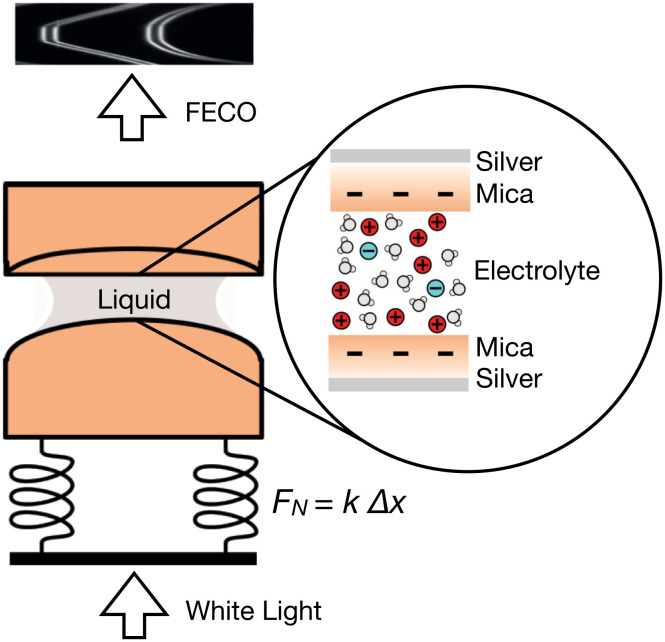
Schematic diagram of the SFB setup showing the path of light through the optical lenses arranged in crossed-cylinder orientation. One of the lenses is mounted on a spring of a known spring constant to allow measurement of normal force across the confined film. A zoom-in to the contact area shows details of the interferometric cavity with the electrolyte confined between two atomically smooth mica sheets of negative charge. The mica surfaces are negatively charged when immersed in the electrolyte.

See *Materials and Methods* below for full details. We used a surface force balance (SFB) to measure the interaction force vs. separation between mica surfaces across films of aqueous solutions, with a spatial resolution of approximately 0.1 nm. The setup is shown schematically in Fig. 2 and has been described in detail previously (28, 29). The mica sheets are freshly cleaved (typical thickness 2 to 3 μm) in a dust-free environment, back-silvered via thermal evaporation, and then mounted on hemicylindrical lenses of radius 1 cm. The lenses are mounted in a crossed-cylinder orientation in the SFB, with one lens on a calibrated leaf spring and the other on a piezo stage. Electrolyte solutions (50 μL) were injected to form a bridging droplet between the two mica-coated lenses. The back-silvered mica–liquid–mica stack creates an optical cavity allowing for interferometric measurement of cavity thickness, and thus mica surface separation, *D*, once the mica thickness has been determined from a contact (*D* = 0) calibration (30). The interferometry involves white light (visible region) directed through the optical cavity and then incident on a grating spectrometer to measure the resulting interference pattern (fringes of equal chromatic order, FECO). The piezo-mounted lens is translated normally, and any force between the two lenses can be determined via deflection of the leaf spring on which the other lens is mounted, yielding distance-resolved intersurface forces. With the radius of lens curvature *R* >  > *D*, the Derjaguin approximation applies, and the measured force, *F*, is related to the interaction free energy per unit area between parallel plates, *E*^||^ by *F*/*R* = 2*πE*^||^. In this way, the measurement provides a high-resolution determination of absolute interaction free energy vs. separation between uniform negatively charged surfaces across the electrolyte.

Random error in measurement of an individual data point within a run is of the order of the size of the point, ∼0.1 nm, and < 0.05 mN/m (no smoothing or averaging is done). Systematic errors between experiments are up to 0.5 nm, < 0.5 mN/m. For this reason, we do not average over many runs, and, instead, present individual examples: Quantities such as oscillatory wavelength and exponential decay length are precisely measured on individual runs and are reproducible between runs, but averaging would lead to “smearing out” these subtle effects. We present repeats of runs and discussion of sources of error in *SI Appendix*.

Mica becomes negatively charged when immersed in water due to dissociation of K^+^ ions from the mineral lattice. The crystal facets used for these experiments are atomically smooth—with no steps in the crystal plane—and uniform in the distribution of negative charge sites. Thus, they provide a highly reproducible model surface for determination of interaction free energy across a medium of interest. Almost all intracellular macromolecules are negatively charged, although charge distribution (e.g., on protein outer surfaces) is irregular. Our experiments provide a systematic and extremely high-resolution method for characterizing the generic features of interactions between negatively charged particles in the electrolyte. However, we note that nonuniformity of charge or topography can also influence biomolecular interactions, and these additional effects will not be captured in the present experiments (31, 32).

Electrolytes were always freshly prepared for each experiment and used within an hour of preparation. Trimethylglycine (TMG, Sigma, > 99%) and potassium chloride (KCl, Alfa Aesar, > 99.995%) were used as received and stored under dry conditions. Samples were prepared by weighing TMG and KCl into a flask and mixing with ultrapure water (Millipore, < 2 ppb total organic content, resistivity 18.2 M*Ω* cm). The refractive indices of solutions were measured using an Abbé Refractometer, allowing for calculation of relevant Hamaker constants; *SI Appendix* for data and discussion.

## Water with Zwitterions Only.

**Fig. 3.**
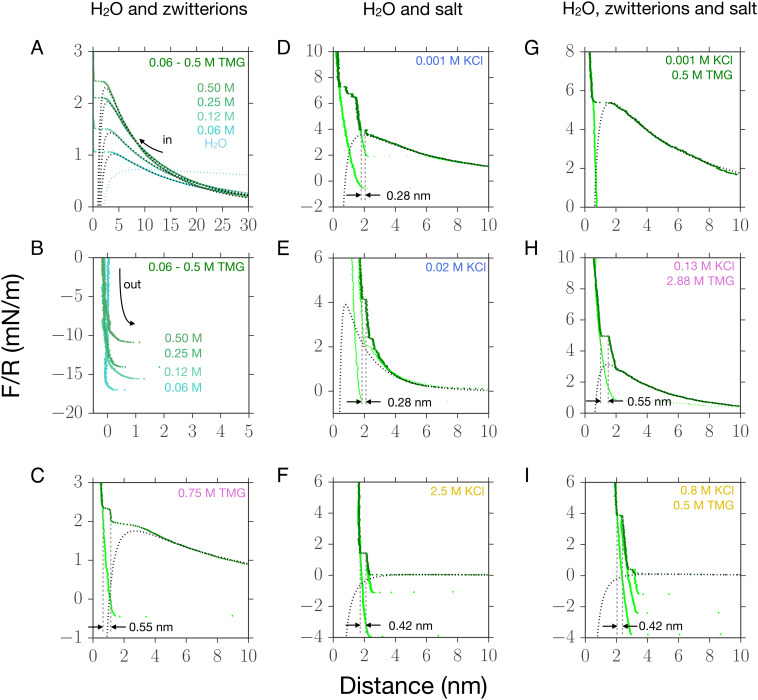
Examples of the interaction force as a function of separation distance measured between negatively charged mica surfaces across electrolyte solutions at varying salt and zwitterion compositions. Approaching force profiles are shown in dark green, and retraction profiles are shown in light green throughout. Black dashed curves are DLVO fits to the data using Eq. **1**; fitting parameters are in *SI Appendix*. (*A*–*C*) Water + TMG at varying concentration of TMG. (*D*–*F*) Water + KCl at varying concentration of KCl. (*G*–*I*) Water + TMG + KCl at concentrations shown in the inset. (*A*) TMG solutions from 0.06–0.50 M cause weak repulsion from large distances, and then, attractive forces dominate below 3 to 4 nm causing instability. A profile for pure water is shown for comparison, from ref. 33. (*B*) Adhesive (negative) forces measured on retraction of surfaces across the same TMG solutions as in (*A*) show weakening adhesion with increasing TMG concentration. (*C*) In 0.75 M TMG steps appear in the force profile, of 0.55 ± 0.05 nm, and on retraction the adhesion is weak. (*D*) 10^−3^M KCl in water shows long-range repulsion, giving a fitted exponential decay length of 8.8 ± 0.5 nm (*P* = 0.80) and then at least five steps of 0.28 ± 0.02 nm at distances below 4 nm. Retractions slow adhesive minima and so outline the overall oscillatory interaction profile. (*E*) 2 × 10^−2^M KCl in water shows double-layer repulsion, fitted decay length 2.5 ± 0.4 nm (*P* = 1.0), followed by steps of 0.28 ± 0.02 nm. (*F*) 2.5 M KCl in water shows a step of 0.42 ± 0.02 nm followed by a hard repulsive wall; on retraction, the minima outline the overall oscillatory profile. (*G*) 10^−3^M KCl + 0.5M TMG in water; long-range repulsion (fitting parameters *κ*^−1^ = 11.8 ± 0.5 nm, *P* = 0.85) of large amplitude; no water layers apparent. (*H*) 0.13M KCl + 2.88M TMG in water; repulsion on approach with steps of 0.55 ± 0.05 nm at distances below 3 nm. (*I*) 0.8M KCl + 0.5M TMG in water; multiple steps of 0.42 ± 0.05 nm on approach of surfaces below 5 nm. Retraction reveals minima of increasing magnitude with decreasing distance. Inset concentration labels are color-coded to indicate the relevant region in the phase diagram, Fig. 4.

Examples of the measured interaction force, as a function of separation distance, between mica sheets across solutions of the zwitterion TMG in water with no added salt are shown in Fig. 3*A*–*C*. The interaction across pure water is also shown for comparison in Fig. 3*A*. For zwitterion concentrations up to 0.5 M (Fig. 3*A*), the surfaces repel from large separations, the repulsion increasing in strength with decreasing separation distance, down to ∼3 nm. At this point, a strong attractive force causes spring instability and the surfaces jump together. The attraction and resulting adhesion of the surfaces are confirmed on separation of the surfaces (Fig. 3*B*), where a finite negative force is required to separate the surfaces from adhesive contact. Increasing the concentration of zwitterions in water, in the range of 0.0 to 0.5 M, leads to stronger repulsion and weaker adhesion.

The general form of these interaction profiles can be compared to predictions from the Derjaguin–Landau–Verwey–Overbeek (DLVO) theory of colloidal stability (1). The DLVO theory supposes that the total interaction force between two particles or surfaces is a sum of their electrostatic and van der Waals interactions. For two identical surfaces interacting across the electrolyte, the electrostatic term is repulsive and arises from the excess osmotic pressure due to overlap of the diffuse layers of countercharge associated with each surface; the charge distribution and thus electrostatic force can be calculated using the Poisson–Boltzmann equation. Here, we choose to solve the Poisson–Boltzmann equation using a linear regulation model for the boundary conditions adapted from Carnie and Chan (34, 35). (*SI Appendix* for a discussion of the model and resulting functional form.) The van der Waals term is attractive and can be calculated using the Hamaker approach (1). Putting these together, the full expression for the force, *F*^DLVO^, is:

[1] $$\begin{array}{cc} \frac{F^{\text{DLVO}}}{R}= & 2\pi E^{\left|\right|}\approx -\frac{A}{6D^{2}}\\ & +4\pi \epsilon_{0}\epsilon \kappa_{D}\psi_{\;\text{eff}}^{2}\frac{e^{-\kappa_{D}D}}{1+\left(1-2p\right)e^{-\kappa_{D}D}}, \end{array}$$

where the approximation is suitable at very low ion concentrations and small diffuse layer potentials (36). Here, *R* is the curvature radius of the crossed cylinders, *A* the mica–electrolyte–mica Hamaker constant, *ϵ*_0_ is the permittivity of free space, *ϵ*_*r*_ is the relative permittivity, *ψ*_eff_ is the effective surface potential, *κ*_*D*_^−1^ is the Debye-Hückel screening length, and *p* is a dimensionless charge regulation parameter ranging from *p* = 0 (constant potential boundary condition) to *p* = 1 (constant charge boundary condition). *κ*_*D*_ is an electrolyte (bulk) property and in a 1:1 electrolyte is as follows:

[2] $$\begin{array}{c} \kappa_{D}^{2}=\frac{2e^{2}\rho }{\epsilon_{r}\epsilon_{0}k_{B}T}, \end{array}$$

where *e* is the electron charge, *ρ* the bulk salt (ion pair) number density, *k*_*B*_ Boltzmann’s constant, and *T* the temperature. In our experiments, *F*, *R*, *D*, *T*, *A*, and *ρ* are measured or controlled, so Eq. **1** can be used to determine *ψ*_eff_ and *κ*_*D*_. This measured (fitted) value of *κ*_*D*_ can also be compared to calculated values from Eq. **2**, which is revealing in respect to *ϵ*_*r*_ as we shall see later.

Fits to Eq. **1** for our measurements with water and zwitterions only, as shown in Fig. 3*A*, show that increasing the concentration of zwitterions leads to an increase in *ψ*_eff_ over the range 43 ± 3 mV to 53 ± 3 mV (*p* = 0.8 ± 0.02). Values of *κ*_*D*_^−1^ for these measurements are in the range 22 ± 2 nm to 13 ± 1 nm (for 0.06 M to 0.5 M TMG), corresponding to a 1:1 salt concentration of approximately 2 to 5 ×10^−4^ M; with no added salt, this small residual ionic strength arises from the autoionization of water, bicarbonate from dissolved CO_2_, and trace acid leached from glassware and from ionization of the mica (28, 37) as well as trace impurities in the TMG samples.

We consider three possible origins of the observed increasing *ψ*_eff_ with increasing zwitterion concentration. First, the zwitterions may adsorb strongly at the mica surface due to their high molecular dipole moment which interacts favorably with the negatively charged surface; the monolayer of zwitterions oriented with positive end at the mica and negative end away presents a more negative effective potential relative to the solution. Notably, even net-neutral zwitterions can modify the effective charge and effective potential at points out of the plane of the zwitterion layer if their dipoles are aligned and thus generating a field in the direction perpendicular to the interface (38). Second, an adsorbed zwitterion layer may increase the absolute charge at the interface by shifting the ionization equilibrium of the mica surface: The positively charged end of the zwitterion can effectively ion-exchange with K^+^ ions from the mica, leading to a higher ionization fraction compared to mica in pure water (39). Third, nonadsorbed zwitterions in the region between the two surfaces will increase the local dielectric permittivity and thus further promote ionization of the mica surfaces.

To distinguish between these mechanisms, we next look to measurements at higher zwitterion concentrations, exemplified in Fig. 3*C* with 0.75M TMG. Here, an additional feature is observed: On approach of the surfaces, we measure a “step” in the force profile, i.e., a steep repulsion which subsequently gives way to attraction, before final contact between the surfaces is reached. The width of the step is 0.55 nm, which corresponds closely to the end-to-end size of the zwitterion molecule, 0.57 nm (40); we suggest that this feature arises from adsorbed layers of zwitterions, with dipoles aligned perpendicular to the interface, at each surface. With this in mind, it is likely that the increasing *ψ*_eff_ with increasing zwitterion concentration arises predominantly from the adsorption of zwitterions at the interfaces, to a greater extent as the zwitterion concentration increases, and the subsequent influence on effective and absolute charge and electrostatic surface interactions. This observation is supported by the reduced adhesion between mica surfaces brought into close contact with increasing zwitterion concentration (Fig. 3*B*); the reduction in adhesion is likely due to steric hindrance, i.e., the adsorbed TMG prevents close contact of the surfaces. Our results resonate with suggestions of dipolar zwitterion alignment and chain formation (41) and are consistent with reports from Govrin et al. (25, 26) showing that the effective surface charge of silica particles also increased with TMG concentration, saturating at around 1 M. Furthermore, we confirm the recent finding of Ridawan et al. that zwitterions form layers at charged surfaces (27).

## Water with Salt Only.

Examples of the measured interaction force between negatively charged surfaces across solutions of KCl in water (with no zwitterion) are shown in Fig. 3*D*–*F*. In 10^−3^ M KCl (Fig. 3*D*), we observe a repulsion from a long range and then, at *D*< 5 nm, alternating repulsive and attractive regions with a wavelength of 0.28 ± 0.02 nm. This oscillatory force—often called a “structural force”—arises from molecular packing between the surfaces and in this case has a wavelength consistent with water-dominated layering. Such water-dominated layering is challenging to measure due to the demanding sensitivity in distance (of about 0.1 nm) and mechanical stability required; however, our measurements here reproduce the early report of Pashley and Israelachvili (42). When the KCl concentration is increased to 20 × 10^−3^ M, Fig. 3*E*, we see that the steps due to force oscillations of wavelength 0.28 nm remain; however, the force required to squeeze out the layers is greater.

In Fig. 3*F*, we show an example at the higher KCl concentration of 2.5 M. The interaction is now dominated by the structural forces in the range below ∼5 nm. The nature of the oscillatory force is also different: In this case of 2.5 M, as for all samples tested above ∼0.5 M, we find a structural lengthscale of 0.42 ± 0.05 nm, likely originating from “salt layering,” due to ion–ion correlations dominating in the near-surface layers (43–45). The negatively charged surface is coated in a layer of cations which overcompensates the surface charge; this is followed by a layer of excess anions, then excess cations, and so on for several layers creating an interfacial region with oscillations between positive and negative charge density away from the surface. This switch from solvent (water)-dominated structural forces at low concentration to salt-dominated structural forces at high concentration is well known to occur in ionic liquids and highly concentrated electrolyte solutions and is closely connected to the Kirkwood line which delineates monotonic from oscillatory decay of charge correlations in bulk electrolyte solutions (46). When surfaces coated in such alternating layers approach one another, electroneutrality is maintained by squeeze-out of an equal number of cations and anions—this results in a structural force wavelength corresponding to a pair of ion layers. To rationalize the observed wavelength of 0.42 ± 0.05 nm, we compare to the repeat distance between close-packed (1,1,1) planes in the KCl unit cell: The distance between two cation planes (or two anion planes) in the rocksalt structure is $a/\sqrt{3}$, with *a* being the lattice constant, giving a repeat distance of 0.36 nm for KCl. Our measured wavelength of 0.42 ± 0.05 nm, being comparable but slightly larger than this crystal-derived value, implies a small swelling of the layers due to the hydration of ions relative to what would be expected for a pure molten salt.

At distances beyond 5 nm, a monotonically decaying force is observed (*SI Appendix*, Fig. S1) but with very weak amplitude. This underscreening has been discussed elsewhere (47) and appears here to have lower amplitude in KCl compared to NaCl (47), perhaps arising from the natural affinity of K^+^ ions for the mica surface, leading to weaker effective charge.

## Water with Zwitterions and Salt.

**Fig. 4.**
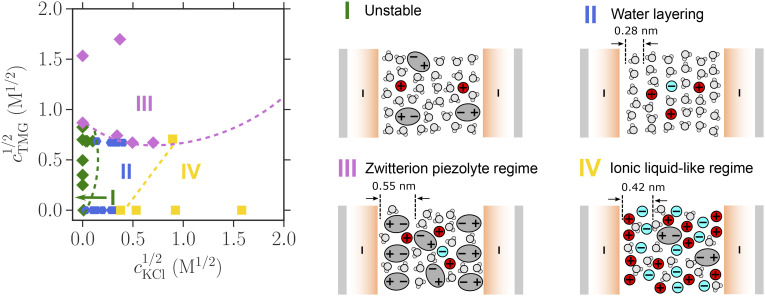
Phase diagram summarizing the range of salt and zwitterion concentrations at which different interfacial forces are observed. (*SI Appendix* for the same data on linear axes.) The points on the plot of TMG concentration versus KCl concentration represent individual experiments, with each experiment involving many force measurements of the sort exemplified in Fig. 3. The behavior at each composition was observed to involve either I) repulsion followed by jump-in, labeled “unstable”; II) oscillatory force due to water-dominated layering; III) repulsive walls due to layers of zwitterions; and IV) oscillatory forces due to salt-dominated layering. Schematic diagrams indicating possible interfacial arrangement of water, ions, and zwitterions are indicated for each.

Interactions across solutions containing both zwitterions and salt are perturbed relative to the salt-only or zwitterion-only cases; in Fig. 3*G*–*I*, we show examples of the measured forces, and in Fig. 4, we construct a phase diagram to delineate the regions in composition for which the behaviors exemplified in Fig. 3 were observed in our experiments. We make the following general observations: i) The presence of zwitterions suppresses the oscillations due to water-layering in low-concentration salt solutions (compare Fig. 3 *D* and *G*, where KCl is at 10^−3^ M in both cases). The resulting smooth repulsive profile is similar to the zwitterion-only case; however, the presence of salt acts to enhance the total repulsive interaction force (compare Fig. 3 *A* and *G*). ii) At high zwitterion concentrations, the zwitterions dominate the near-surface layering (see 0.55 nm layers in Fig. 3*H*). iii) At high salt concentrations, salt-layering is observed (see 0.42 nm layers in Fig. 3*I*), resulting in an oscillatory interaction profile extending up to 5-nm separation.

Fig. 4 shows the electrolyte compositions, in terms of salt and zwitterion concentration, at which these distinct characteristics dominate the overall interaction. We also provide diagrams based on our measured layer dimensions and interaction forces, indicating how the near-surface molecular arrangements are likely to contribute to these outcomes.

**Fig. 5.**
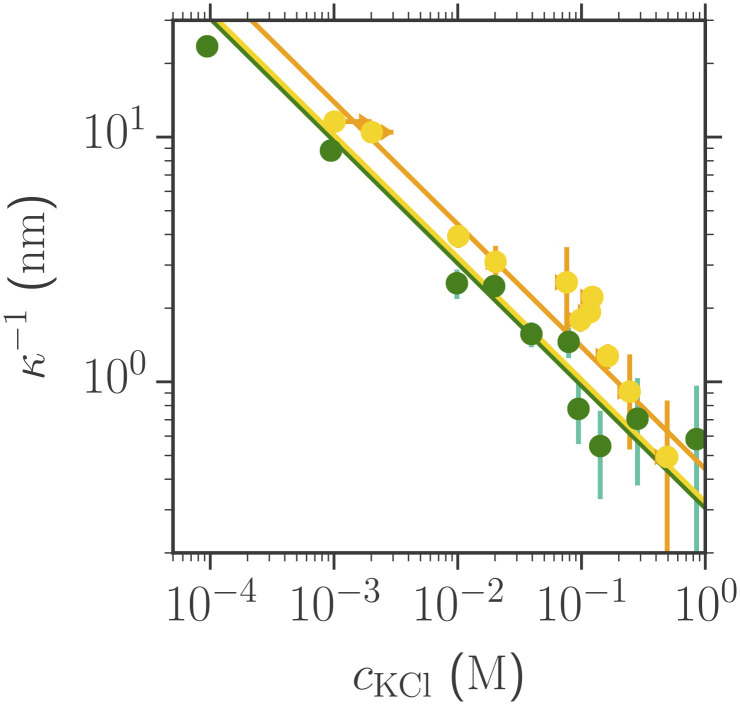
Exponential decay lengths of the double-layer repulsion as a function of KCl salt concentration with and without zwitterions. Green points: Measurements with water + KCl only. Yellow points: Measurements with water + KCl + 0.5 M TMG. Also shown for comparison are calculations of screening length vs. concentration for various *ϵ*_*r*_: the green line indicates the Debye-Hückel prediction from Eq. **2** using *ϵ*_*r*_ = 78.5; the yellow line indicates the Debye-Hückel prediction using *ϵ*_*r*_ = 87.9; and the orange line indicates fitted decay length vs. concentration for solutions containing water + salt + 0.5 M TMG, yielding a fitted *ϵ*_*r*_ = 163 ± 10 for the zwitterion-containing solutions.

The influence of zwitterions on forces across electrolyte solutions is also detected in the longer-range interaction. By fitting the monotonic tail of the interaction forces to the electrostatic repulsion term in Eq. **1**, the decay length, *κ*^−1^, can be extracted. In very dilute solutions of the electrolyte, *κ*^−1^ is expected to match the Debye-Hückel prediction, i.e., *κ* = *κ*_*D*_, but at higher salt concentrations, the decay length is known to deviate from mean field predictions (48). Here, it is of interest whether the presence of zwitterions has an influence on the measured decay length. In Fig. 5, we show the measured screening lengths in solutions of water and KCl as a function of salt concentration, comparing solutions without TMG present (green points) and with 0.5M TMG (yellow points). There is a systematic increase in the screening length of ∼50% when zwitterions are present in the electrolyte solution. The increase cannot be simply explained by the small linear dielectric decrement relevant at low solute concentration, i.e., *ϵ*_*r*_^*calc*^ = *ϵ*_*r*_^0^ + (*δ* × *c*_*TMG*_) (49), with *ϵ*_*r*_^0^ being the relative permittivity of the pure solvent and *δ* the dielectric decrement, predicting an extrapolated value of *ϵ*_*r*_^*calc*^ = 87.9 for 0.5M TMG; compare the green and yellow lines in Fig. 5. Instead, adjusting the dielectric constant to fit the measurements results in *ϵ*_*r*_^eff^ = 163 ± 10 (orange line). This enormous increase in the effective dielectric constant for solutions containing 0.5 M TMG resonates with recent experiments by Govrin et al. (25, 26), where the decay length of zwitterion-containing solutions was also found to be larger than predicted. Furthermore, our observations here build on recent dielectric spectroscopy measurements by Mei et al. (50) showing that zwitterions increase the solution dielectric constant more rapidly than expected from a simple molar ratio of dielectric constants of the solvent and zwitterion. They propose that zwitterions act as “dielectricizers” due to their large polarizability volume; in particular, at low and mid concentrations, the zwitterions have a strong polarizing effect on surrounding water and consequently have a stronger influence on the polarization response of the solution than anticipated. Our observation that *ϵ*_*r*_^eff^ for 0.5M TMG in water is approximately double the value of pure water resonates with the results of Mei et al., where similar enhancements were observed for a range of zwitterions in ethylene glycol (50). Reflecting on the general form of Eq. **1** as a framework for interpreting interactions across the electrolyte solutions studied, we see that addition of zwitterions influences all terms: The van der Waals term decreases very slightly, while the electrostatic term increases in both its magnitude (prefactor) and range (exponential decay length). We have interpreted the enhanced magnitude as arising from an increased surface charge and the increase of decay length as due to the enhanced dielectric permittivity. Taken together, the effect of zwitterions is to increase colloidal stability of charged bodies through these several mechanisms.

## Zwitterions Tune Interactions Across Electrolytes.

Our model experiments interrogating the effect of a single salt and zwitterion combination on interaction forces between charged surfaces reveal a subtle pallet of stabilizing strategies beyond the mean-field DLVO interactions often assumed to determine biomolecular interactions in solution. In particular, we make the following conclusions on the role of zwitterions in modifying interparticle forces compared to simple salt solutions: 1) Zwitterions adsorb strongly at charged surfaces, creating monolayers or multilayers—depending on the zwitterion concentration—which enhances the effective surface charge and thus the magnitude of repulsive interactions between particle surfaces. 2) Zwitterions frustrate the regular near-surface ordering of water molecules, disrupting the oscillations in surface interactions that would extend to about 5 nm in the absence of zwitterions. This results in a smoothed interaction potential between the surfaces at mid range. 3) In electrolytes containing both zwitterions and salt, the interfacial structure is delicately influenced by the relative composition; either zwitterion layers or salt layers can form. In either case, the surfaces experience enhanced repulsion, i.e., stability against collapse: Zwitterions, as well as salt, can provide a stabilization strategy for particles at high hydrostatic pressure. 4) Despite being net-neutral, zwitterions increase the decay length of electrostatic interactions in water compared to the Debye-Hückel prediction; the observation confirms recent observations of the dielectricizer effect of zwitterions and is not simply accounted for by the small change in dielectric permittivity of the electrolyte anticipated by simple linear mixing.

Overall, our measurements contribute to understanding the simultaneous tuning of intracellular interactions and osmoregulation (6, 14). Even in a simple three-component electrolyte, particle interactions are complex and far beyond the interpretations of mean-field models such as the DLVO theory. The results have broad implications in nature and technology: from providing a mechanistic understanding of the piezolytic and osmolytic roles of zwitterions in marine biochemistry to informing development of halo-tolerant crop species in response to anthropogenic accumulation of salts in agricultural soil (51–53).

## Materials and Methods

### Materials.

Optical-grade mica (ruby muscovite, S&J Trading VI special grade) was cleaved and cut using established protocols. Facets of constant thickness (identified by their single interference color) were attached to a freshly cleaved mica substrate and subsequently coated with a layer of silver by evaporating silver shot (99.9999%, Alfa Aesar) using an HHV Auto306 (HVV Ltd) thermal evaporator. Silver layers were typically ∼40 nm thick. Silver-coated mica sheets were stored in vacuum until use. Prior to measurement, substrates were removed from vacuum, and small (< 1 cm^2^) facets were cut and attached to fused silica cylindrical lenses (Knight Optical) using EPON 1004 (Shell) glue. Lenses were mounted in the SFB in a crossed-cylinder orientation, and osmolyte solutions were injected between them to form a capillary bridge. The bottom lens holder had a capacity of around 2 ml, so enough liquid was added to reach the edge of the lenses so that any further addition of liquid would be in contact with liquid held between the two lenses. In some instances, further osmolyte solutions were injected in situ to dilute one of the solutes during the measurement.

## Supplementary Material

Appendix 01 (PDF)

## Data Availability

All raw data (force and distance data points) are available from the Oxford Research Archive, https://doi.org/10.5287/bodleian:BROE4obpd.
