# Supplementary material for: Zwitterions fine-tune interactions in electrolyte solutions
Source: Proc Natl Acad Sci U S A. 2023 Feb 14;120(8):e2215585120. doi: 10.1073/pnas.2215585120 (PMC9974414; doi:10.1073/pnas.2215585120)
Supplement: Supplementary file 1 — Appendix 01 (PDF) [file pnas.2215585120.sapp.pdf]

Supporting Information for

Zwitterions fine-tune interactions in electrolyte solutions

James E. Hallett, Kieran J. Agg and Susan Perkin

Susan Perkin
E-mail: susan.perkin@chem.ox.ac.uk

This PDF file includes:

Supporting text
Figs. S1 to S6
Tables S1 to S2
SI References

Supporting Information Text

Screening at high salt concentration

Here we provide example force profiles for a concentrated salt system, showing evidence for a long-range repulsive force of greater range than expected from Debye-Hückel screening (equation 2). Figure S1 shows the same data as for figure 3f in the main text but in log-linear representation. A long range exponential-like repulsion is shown, approximately an order of magnitude weaker than at lower salt concentrations (c.f. figure S3).

Experimental error and repeated measurements

In Figure 3 of the main text we choose to display individual measurement runs because this shows most clearly the small features, such as steps that occur when a molecular layer is squeezed out. Such features are visible because the random error in measurement of an individual data point within a run is very small (and of the order of the size of the points in Fig.3), ca. 0.1nm , $<$ 0.05 mN/m ; no smoothing or averaging is done. When we make repeated measurements using different mica sheets, different solutions, different setup/alignment of the apparatus then we tend to observe systematic error between measurements of up to 0.5nm , $<$ 0.5 mN/m. This arises from small errors in optical alignment; error in measurement of radius of curvature, R (5%), spring constant, K, (5%), error in temperature, T, (about 0.1K), and error in concentration of samples. Quantities such as oscillatory wavelength and exponential decay length are extremely precisely measured on individual runs, and are also highly reproducible between runs, but averaging would 'smear out' these subtle effects. Here in Figures S1-S5 we show examples from some of our experimental conditions of repeated runs, illustrating the above.

In figures S1-S5 we provide examples of repeated measurements of force profiles, including measurements made on different days with freshly made solutions, new optical alignment, etc. In general we find that the overall form of the force profiles appears to have no dependence on equilibration time (between adjacent runs), or time after injection (from start of the experiment). Small differences between runs include some variation in squeeze-out force of confined layers (see e.g. figures S3, S4 and S5), and non-systematic variation in the magnitude of long-range electrostatic interactions (figure S2). However, layer thicknesses and screening lengths are highly reproducible. Variations between runs or separate experiments can be attributed to e.g. inhomogeneities in the curvature of the lenses (when measuring on different spots during the same experiment), thermal or mechanical drift in the optical path, or molecular rearrangements at the mica surface.

Phase behaviour

Here we provide an alternative representation of figure 3 from the main text, with linear axes rather than square root (figure S6).

Fit parameters

In table S1 we provide details of the parameters used with equation 1 (main text) to yield the fits shown in figure 3.

Hamaker constants between mica surfaces

Here we estimate Hamaker constants between mica surfaces across electrolyte solutions for a number of compositions using equation 1 (1). Here, k is the Boltzmann constant; T is the temperature (295 K); ϵ_m and ϵ_e are the dielectric constants of mica and electrolyte, respectively; h is the Planck constant; ν_e is the plasma frequency of the free electron gas; and n_m and n_e are the refractive indices of mica and electrolyte, respectively. The frequency of the free electron gas was taken as 3×10^{15} s^{-1} and the refractive index of mica was taken as 1.6. The refractive indices of electrolyte solutions were measured using an Bellingham and Stanley Abbe 60 Refractometer and a sodium lamp of wavelength 589.3 nm. Measured values are shown in table S2.

$$A = \frac{3}{4}kT\left(\frac{\epsilon_m - \epsilon_e}{\epsilon_m + \epsilon_e}\right)^2 + \frac{3h\nu_e}{16\sqrt{2}}\frac{(n_m^2 - n_e^2)^2}{(n_m^2 + n_e^2)^{\frac{3}{2}}} \quad [1]$$

The dielectric constant of the electrolyte was estimated using the following expression:

$$\epsilon_e = \epsilon_r^0 + \delta_{\mathrm{KCl}}c_{\mathrm{KCl}} + \delta_{\mathrm{TMG}}c_{\mathrm{TMG}} \quad [2]$$

where ϵ_r^0 is the dielectric constant of water, δ is the dielectric decrement and c is concentration. We have taken δ_{TMG} to be 18.2/M and δ_{KCl} to be -8.85/M, following Edsall *et al.* (2) and Chen *et al.* (3) respectively.

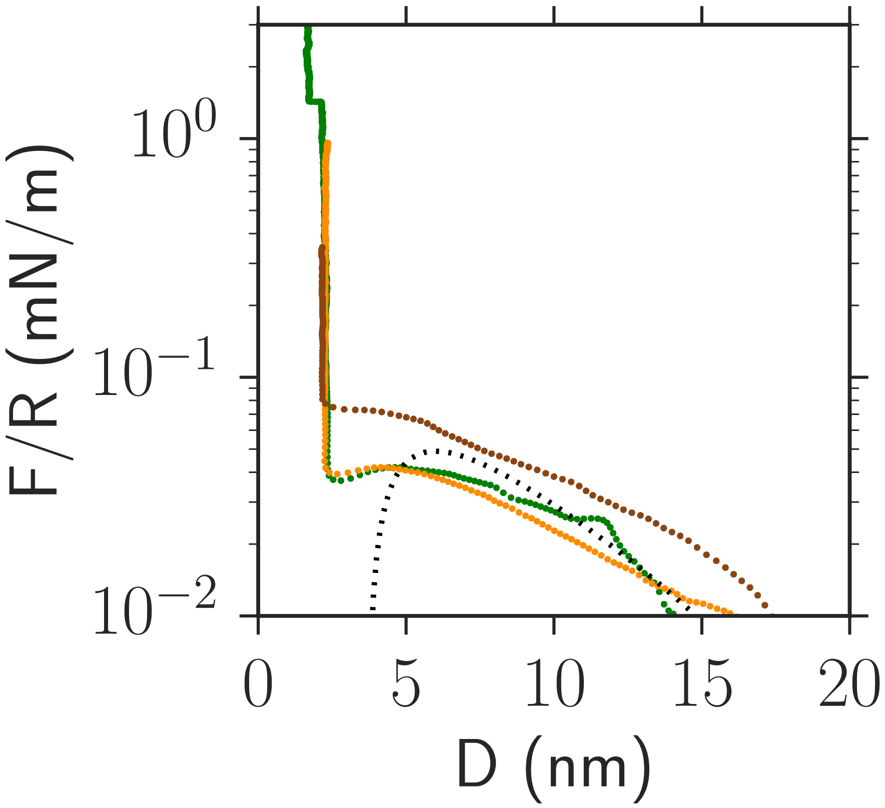

Fig. S1. Interaction force as a function of separation distance measured between negatively charged mica surfaces across 2.5 M KCl electrolyte solution. Approaching force profile is shown in dark green and repeat measurements of the long range force are shown in orange and brown. Black dashed curves are DLVO fits to the data using equation 1 (main text), with a fitted decay length of 5.5 $\pm$ 1.2 nm (p=0.70) and surface potential of 14 $\pm$ 6 mV.

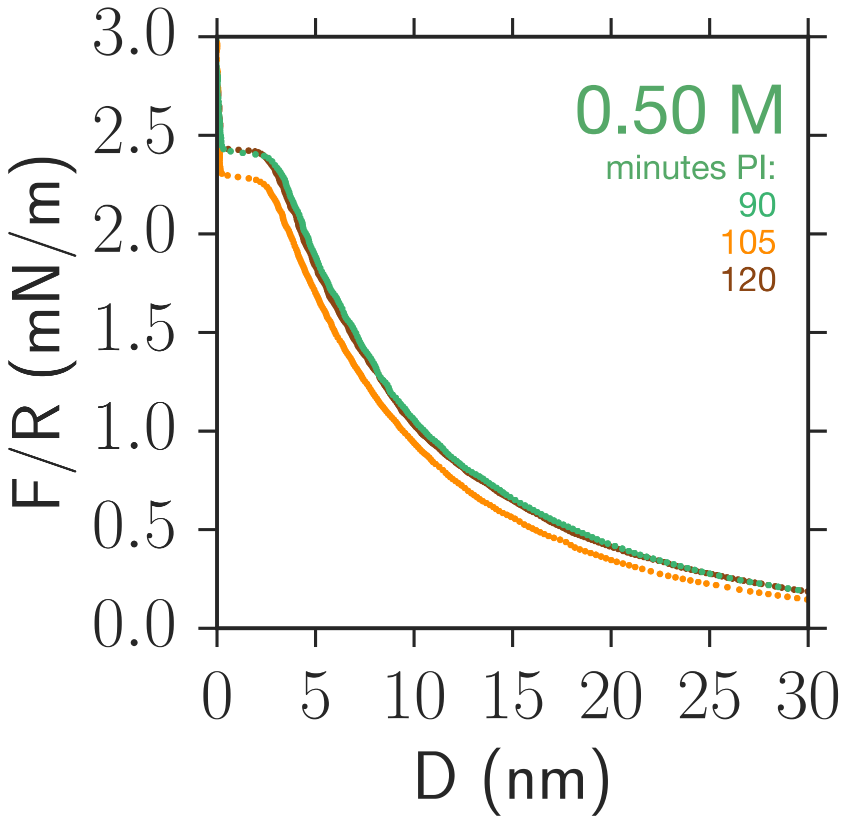

Fig. S2. Interaction force as a function of separation distance measured between negatively charged mica surfaces across 0.5 M TMG solution. Times are given post injection (PI) of the TMG solution between the mica surfaces.

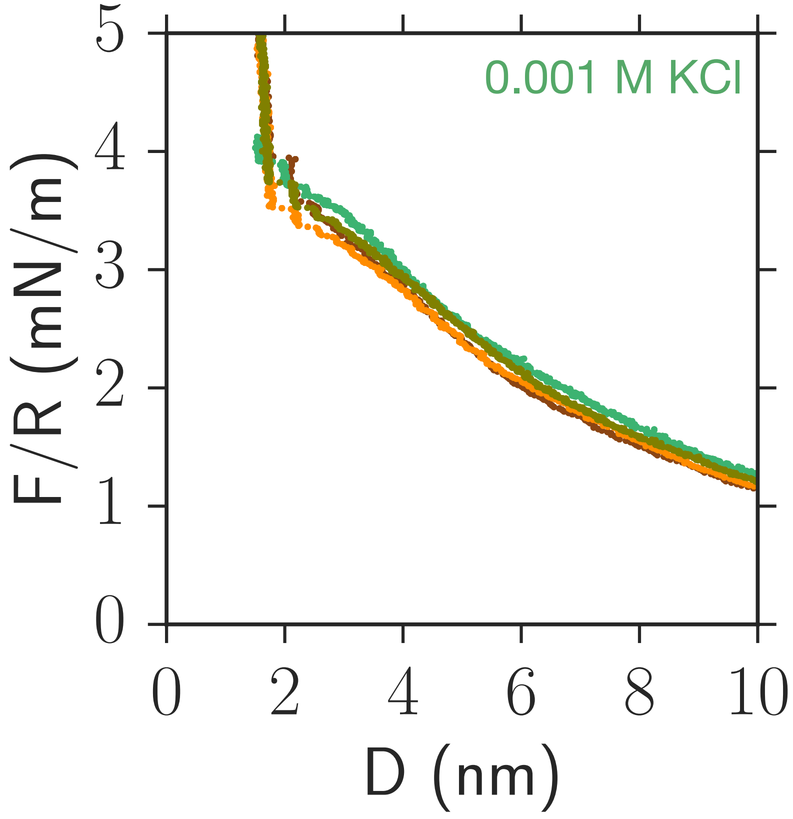

Fig. S3. Interaction force as a function of separation distance measured between negatively charged mica surfaces across 0.001 M KCl solution, showing type II structure, i.e. water layering. Repeated measurements are shown in different colours.

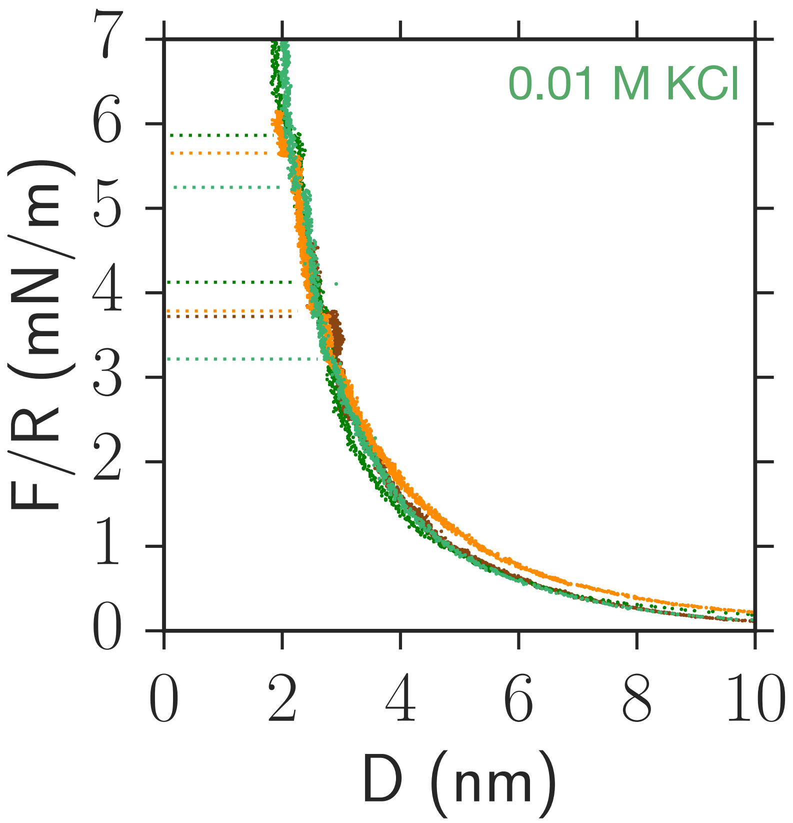

Fig. S4. Interaction force as a function of separation distance measured between negatively charged mica surfaces across 0.01 M KCl solution, showing type II structure, i.e. water layering. Repeated measurements are shown in different colours. Dotted lines indicate surface force at which water layers are squeezed out on repeated measurements and are colour coded to match their corresponding force curve.

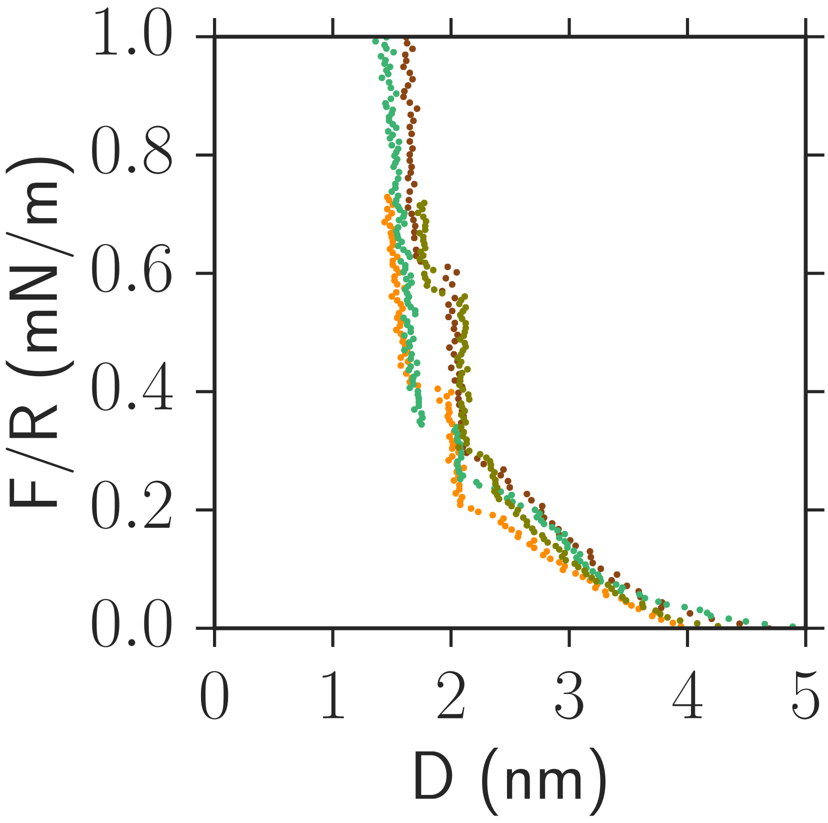

Fig. S5. Interaction force as a function of separation distance measured between negatively charged mica surfaces across 0.098 M KCl, 0.45 M TMG solution, showing type II structure, i.e. water layering. Repeated measurements are shown in different colours.

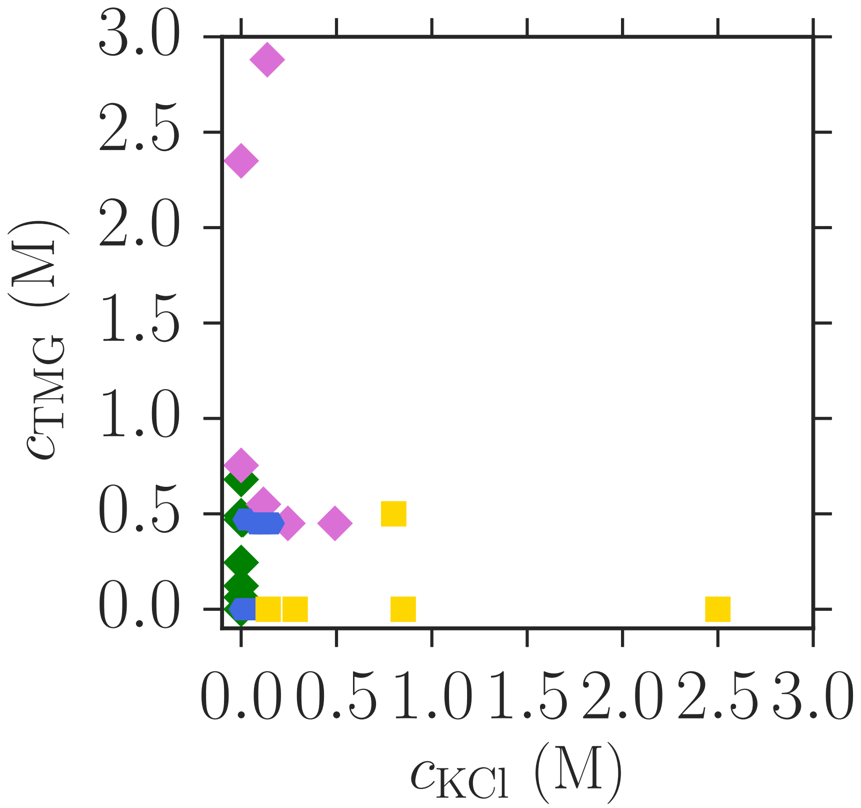

Fig. S6. Linear-linear representation of figure 3 from the main text: Phase diagram summarising the range of salt and zwitterion concentrations at which different interfacial forces are observed. The points on the plot of TMG concentration versus KCl concentration represent individual experiments; each experiment involving many force measurements of the sort exemplified in Fig. 3. The behaviour at each composition was observed to involve either (green) repulsion followed by jump-in, labelled "unstable"; (blue) oscillatory force due to water-dominated layering; (pink) repulsive walls due to layers of zwitterions; (yellow) oscillatory forces due to salt-dominated layering.

Table S1. Table of parameters used with equation 1 for fits shown in figure 3. Concentrations of potassium chloride and trimethyl glycine are (c_{KCl} and c_{TMG}), the fitted screening length κ_D^{-1}, charge regulation parameter p, fitted surface potential ψ_{eff} and Hamaker constant A are shown.

Figure	c_{KCl} (M)	c_{TMG} (M)	κ_D^{-1} (nm)	p	ψ_{eff} (mV)	A ($\times 10^{-20}$J)
a	0.000	0.06	22.2	0.79	43.3	2.06
a	0.000	0.13	19.9	0.81	46.8	2.05
a	0.000	0.25	14.9	0.79	50.6	2.02
a	0.000	0.50	13.0	0.81	49.4	1.97
c	0.000	0.75	19.9	0.88	43.5	1.91
d	0.001	0.00	8.8	0.80	53.9	2.08
e	0.020	0.00	2.5	1.00	31.4	2.08
f	2.500	0.00	5.5	0.69	14.5	1.76
g	0.001	0.47	11.8	0.86	62.0	1.97
h	0.130	2.88	5.2	0.82	41.5	1.47
i	0.800	0.50	8.6	0.96	13.5	1.80

Table S2. Table of Hamaker constants estimated for a number of different TMG-KCl compositions. Concentrations of potassium chloride and trimethyl glycine are (c_{KCl} and c_{TMG}), refractive index n, dielectric constant ϵ_e and Hamaker constant A are shown.

c_{KCl} (M)	c_{TMG} (M)	n	ϵ_e	A ($\times 10^{-20}$J)
0.00	0.00	1.3323	78.4	2.08
0.00	1.00	1.3490	96.6	1.86
1.00	0.00	1.3419	69.4	1.94
1.00	1.00	1.3585	87.7	1.73
2.50	0.00	1.3551	56.2	1.76
0.13	2.80	1.3814	128.2	1.47

References

1. JN Israelachvili, *Intermolecular and Surface Forces.* (Academic Press), Third edition, (2011).
2. JT Edsall, J Wyman Jr, Studies in the physical chemistry of betaines and related substances. i. studies of dielectric constants and apparent molal volume1. *J. Am. Chem. Soc.* **57**, 1964–1975 (1935).
3. T Chen, G Hefter, R Buchner, Dielectric spectroscopy of aqueous solutions of kcl and cscl. *The J. Phys. Chem. A* **107**, 4025–4031 (2003).
